# Preemptive perineural bupivacaine attenuates the maintenance of mechanical and cold allodynia in a rat spinal nerve ligation model

**DOI:** 10.1186/s12871-015-0113-x

**Published:** 2015-10-06

**Authors:** John L. Clifford, Alberto Mares, Jacob Hansen, Dayna L. Averitt

**Affiliations:** Pain Management Research Area, United States Army Institute of Surgical Research, Fort Sam Houston, TX USA; Department of Biology, Texas Woman’s University, PO Box 425799, Denton, TX 76204-5799 USA

**Keywords:** Neuropathic, Pain, Allodynia, Hyperalgesia, Bupivacaine, Preemptive

## Abstract

**Background:**

Neuropathic pain is evasive to treat once developed, however evidence suggests that local administration of anesthetics near the time of injury reduces the development of neuropathic pain. As abnormal electrical signaling in the damaged nerve contributes to the initiation and maintenance of neuropathic pain, local administration of anesthetics prior to injury may reduce its development. We hypothesized that local treatment with bupivacaine *prior to* nerve injury in a rat model of spinal nerve ligation (SNL) would attenuate the initiation and/or maintenance of neuropathic pain behaviors.

**Methods:**

On the day prior to SNL, baseline measures of pre-injury mechanical, thermal, and/or cold sensitivity were recorded in adult male Sprague–Dawley rats. Immediately prior to SNL or sham treatment, the right L5 nerve was perineurally bathed in either 0.05 mL bupivacaine (0.5 %) or sterile saline (0.9 %) for 30 min. Mechanical allodynia, thermal hyperalgesia, and/or cold allodynia were then examined at 3, 7, 10, 14 and 21 days following SNL.

**Results:**

Rats exhibited both mechanical and cold allodynia, but not thermal hyperalgesia, within 3 days and up to 21 days post-SNL. No significant pain behaviors were observed in sham controls. Preemptive local bupivacaine significantly attenuated both mechanical and cold allodynia as early as 10 days following SNL compared to saline controls and were not significantly different from sham controls.

**Conclusions:**

These data indicate that local treatment with bupivacaine prior to surgical manipulations that are known to cause nerve damage may protect against the maintenance of chronic neuropathic pain.

## Background

It is estimated that 100 million Americans suffer from chronic pain [1]. Chronic *neuropathic* pain is a common complication that develops in many patients following surgery involving intraoperative nerve damage [2]. Military Service Members represent a subset of the population that is especially at risk. Approximately 44 % of the Service Members deployed to either Iraq or Afghanistan [3] and over 80 % of veterans report chronic pain [4]. Over half sustain injuries involving nerve damage, such as motor vehicle accidents, blasts and burns, and lower back injuries [5–7]. A subset is subject to amputations, nerve dissections and invasive surgeries involving further nerve damage that may exacerbate the development of neuropathic pain [8]. As neuropathic pain is elusive to treat after it has developed, treatment strategies that effectively reduce the incidence of neuropathic pain are desired.

Electrical hyperexcitability and abnormal nerve pulse generation occur in injured sensory neurons [9–11]. The behavioral hallmark of this altered neuronal signaling is abnormal responsiveness to non-noxious stimuli, termed allodynia. Preclinical research indicates that if early spontaneous afferent activity following nerve damage is blocked by local anesthetics, allodynia may not develop [12]. This report, along with others using a range of neuropathic pain models [12–15], indicates a critical treatment window to reduce the probability of developing a chronic neuropathic pain condition. An attempt at local anesthesia during or immediately following injury has been postulated as a treatment strategy. Indeed, a recent advance in pain management in wounded Service Members is the use of regional anesthesia on the battlefield [16–18].

Many studies have characterized the effects of anesthetics on treatment immediately following nerve damage, as treatment is often sought *following* injury. However, local preemptive anesthetic treatment may be applied in instances where nerve damage is imminent, such as invasive surgery or amputation, however the effects of preemptive versus postoperative analgesia on the development of chronic neuropathic pain are unclear [19]. It may be possible to directly prevent various changes in primary afferents, spinal sites and descending modulation *prior to* nerve insult. In support, preemptive blockade of peripheral afferent firing prior to oral surgery significantly reduces pain ratings and central sensitization 48 h following surgery [20]. It is then possible that preemptive local anesthetic treatment can prevent the development of chronic neuropathic pain conditions.

The anesthetic bupivacaine blocks nerve impulses by binding to the intracellular portion of voltage-gated sodium channels to prevent depolarization and is currently approved for clinical use as a long-duration peripheral nerve block [21]. We hypothesized that local preemptive bupivacaine treatment prior to nerve injury in a rat model of spinal nerve ligation (SNL) would attenuate the initiation and/or maintenance of neuropathic pain behaviors. Our results provide preclinical evidence of the analgesic effect of a single perineural application of bupivacaine prior to nerve injury on neuropathic pain outcomes over 3 weeks post-SNL. Bupivacaine used as a preemptive treatment may prevent or attenuate the occurrence of chronic neuropathic pain conditions in Service Members and other patient populations.

## Methods

### Subjects

A total of 44 adult (200–300 g) intact male Sprague–Dawley rats (Charles River Laboratories, Wilmington, MA, USA) were used in these experiments. Rats were pair housed in a 12:12 h light:dark cycle with ad libitium access to food and water. All studies were approved by the U.S. Army Institute of Surgical Research Institutional Animal Care and Use Committee and conform to federal guidelines and guidelines of the International Association for the Study of Pain. This study was conducted in strict compliance with the Animal Welfare Act, implementing Animal Welfare Regulations, and the principles of the Guide for the Care and Use of Laboratory Animals. The animal facility is fully accredited by the Association for the Assessment and Accreditation of Laboratory Animal Care, International (AAALAC, Intl.).

### Drugs

Forane (isoflurane, USP) was purchased from Baxter Healthcare (Deerfield, IL, USA) for use as volatile gas anesthesia (3 % for induction; 2–2.5 % for maintenance). Bupivacaine hydrochloride, USP (5 mg/mL; 0.5 %) was purchased from Hospira (Lake Forest, IL, USA).

### Spinal nerve ligation and preemptive bupivacaine treatment

Animals were anesthetized with isoflurane delivered via a rat-specific nose cone and placed in the prone position. The paraspinal muscles were separated from the spinous processes at the L4-S2 levels. The right L6 transverse process was removed to allow identification of the L4 and L5 spinal nerves. Prior to ligation, the right L5 spinal nerve was bathed in either 0.05 mL bupivacaine (*n* = 16) or 0.9 % sterile saline (*n* = 16), by pipetting directly onto the exposed nerve, and allowed to remain for 30 min before ligation occurred. A non-absorbable 6–0 silk suture was then tied around the right L5 spinal nerve, being careful not to damage L4, as previously characterized [22]. The fascia was sutured using 3–0 silk suture and the skin closed with wound clips. Silver sulfadiazine ointment (1 %; Watson) was applied to the incision site immediately following surgery. Sham animals (*n* = 12) received all the same surgical manipulations without the actual tying of the ligation. Rats were allowed to recover for 3 days prior to behavior testing.

### Mechanical allodynia

**Fig. 1:**
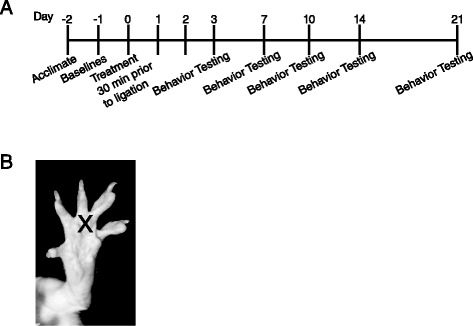
Experimental Design. Timeline illustrating the experimental design by day pre- or post-spinal nerve ligation surgery (**a**). Image illustrates as marked by X the location of application of the non-noxious mechanical, noxious thermal, and non-noxious cold stimuli at the plantar surface of the rat hindpaw (**b**)

To measure changes in sensitivity thresholds to non-noxious mechanical stimulus, 23 rats (*n* = 8 SNL and saline treated; *n* = 8 SNL and bupivacaine treated; *n* = 7 sham) were acclimated to the testing apparatus for 20 min. A Dynamic Plantar Aesthesiometer (Ugo Basile; Collegeville, PA, USA) was used to assess the force (in grams) required to elicit a paw withdrawal from a blunt mechanical stimulus, as previously described [23]. For this test, rats were placed in a Plexiglass box on an elevated grid platform and a blunt mechanical probe was aimed at the base of the third and fourth toe on the hindpaw (Fig. 1b). This location was chosen based on previous reports that the highest sensitivity to mechanical stimuli following SNL is observed at this location [24]. The force of the mechanical stimulus was increased with a ramp of 3 g/s over 10 s with a cutoff of 30 g to avoid mechanical lifting of the paw by the device. The average force required to elicit a paw withdraw over 3 trials was recorded. Baseline measurements of force required to elicit paw withdrawal from both the ipsilateral and contralateral hindpaws were recorded 24 h prior to nerve ligation as a measure of pre-injury mechanical sensitivity. Mechanical allodynia was then assessed at 3, 7, 10, 14 and 21 days following spinal nerve ligation (Fig. 1a). This time course was chosen based on previous reports [22, 25]. The behavioral analyst was blinded to the experimental group of each animal tested.

### Thermal hyperalgesia

A subset of the same rats used to examine mechanical allodynia were also used to examine changes in sensitivity thresholds to a noxious thermal stimulus (*n* = 6 SNL and saline treated; *n* = 7 SNL and bupivacaine treated; *n* = 3 sham). Paw withdrawal latencies to a thermal stimulus were determined using the Plantar Test Apparatus (Hargreaves Method, IITC) as previously described [26]. For this test, rats were placed in a clear Plexiglass box resting on an elevated glass plate and allowed to acclimate for 20 min. A radiant beam of light was then aimed at the plantar surface of the hindpaw and the average paw withdrawal latency over 3 trials was recorded. Baseline measurements of paw withdrawal in seconds from both the ipsilateral and contralateral hindpaws were recorded 24 h prior to nerve ligation as a measure of pre-injury thermal sensitivity. Thermal hyperalgesia was then assessed at 3, 7, 10, 14 and 21 days following nerve ligation. A period of 4 h was retained between mechanical allodynia and subsequent thermal hyperalgesia testing to prevent behavioral sensitization of paw withdrawal responses to the stimuli.

### Cold allodynia

A separate group of rats was used to examine nocifensive behaviors to a cold stimulus. Rats (*n* = 8 SNL and saline treated; *n* = 8 SNL and bupivacaine treated; *n* = 5 sham) were placed in a Plexiglass box resting on an elevated grid platform and a stream of either 100 μL acetone (Fisher Scientific) or 0.9 % sterile saline (room temperature) was applied to the plantar surface of the hindpaw, as previously described [27]. The number of shakes and/or licking episodes of the ipsilateral and contralateral hindpaws due to the cooling evoked by acetone evaporation [28] was measured over a 2 min observation period. The mean of 2 consecutive trials with a 5 min interval was calculated as a measure of nocifensive behavior to a cold stimulus. Cold allodynia was then assessed at 3, 7, 10, 14 and 21 days following spinal nerve ligation.

### Statistical analysis

Data are expressed as mean ± standard error of the mean force to withdrawal in grams, paw withdrawal latencies in seconds, or number of nocifensive responses to acetone application. Data were analyzed by two-way repeated measures ANOVA across all time points. Individual groups were compared using Tukey’s multiple comparisons *post hoc* analysis. The statistical significance was tested at *p* < 0.05. All data were analyzed using GraphPad Prism software version 6 (GraphPad).

## Results

All rats were acclimated to the testing apparatuses two days prior to spinal nerve ligation. Baseline measures of mechanical, thermal, and/or cold sensitivity were recorded 24 h prior to SNL (Fig. 1a) to determine pre-injury thresholds. To examine changes in sensitivity, non-noxious mechanical, noxious thermal and non-noxious cold stimuli were applied to the mid-plantar surface (Fig. 1b) of the right (ipsilateral) and left (contralateral) hindpaws. SNL-evoked neuropathic pain behaviors were examined on 3, 7, 10, 14 and 21 days following preemptive local treatment (bupivacaine, saline, sham). There was a significant main effect of treatment [F(2,20) = 16.91; *p* < 0.0001] and time post-injection from baseline [F(5100) = 12.52; *p* < 0.0001] on the degree of SNL-evoked mechanical allodynia.

**Fig. 2:**
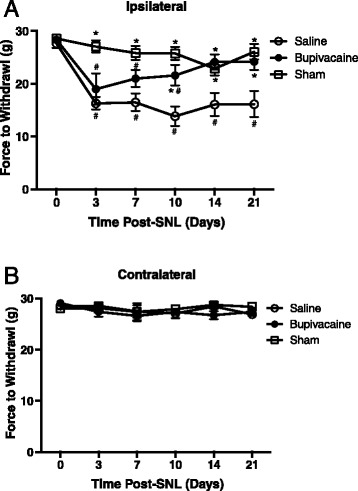
Preemptive bupivacaine attenuates mechanical allodynia evoked by spinal nerve ligation. Force in grams required to elicit hindpaw withdrawal from a non-noxious mechanical stimulus applied to the ipsilateral (**a**) and contralateral (**b**) hindpaw. Responses were recorded prior to and 3, 7, 10, 14 and 21 days following bupivacaine (*n* = 8) or saline (*n* = 8) treatment prior to spinal nerve ligation. A sham (*n* = 7) group was also tested. Asterisks denote significant main effect of treatment detected by two-way ANOVA and Tukey’s posthoc analysis. * denotes significance from saline treated at *p* < 0.05; # denotes significance from baseline at *p* < 0.05

In rats that received a 30-min perineural application of saline at the L5 spinal nerve prior to nerve ligature, the ipsilateral hindpaw displayed significant mechanical allodynia within 3 days and lasting up to 21 days compared to sham controls and baseline (Fig. 2a). In rats that received a preemptive 30-min perineural application of bupivacaine, mechanical allodynia was significantly attenuated at 10, 14 and 21 days following SNL as compared to saline treated controls (Fig. 2a). Furthermore, there was a significant interaction of treatment over time from baseline [F(10,100) = 2.94; *p* = 0.0028]. The 3, 7 and 10 day time points were significantly lower than baselines (*p* < 0.05), however the 14 and 21 day time points were not significantly different from baseline (*p* > 0.05). All time points following saline treatment were significantly lower than baseline measures (*p* < 0.05). Mechanical allodynia in rats treated with bupivacaine was not significantly different from sham controls as early as 7 days post-SNL (*p* > 0.05). There was no significant main effect of time [F(5100) = 1.26; *p* = 0.29] or treatment [F(2,20) = 0.48; *p* = 0.62] in the contralateral hindpaw (Fig. 2b).

**Fig. 3:**
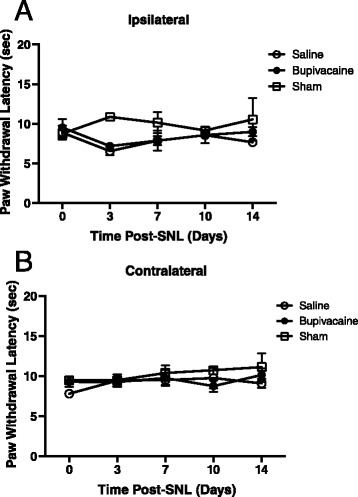
No effect of spinal nerve ligation or bupivacaine on thermal hyperalgesia. Paw withdrawal latency to a noxious thermal stimulus applied to the ipsilateral (**a**) and contralateral (**b**) hindpaw. Responses were recorded prior to and 3, 7, 10, 14 and 21 days following bupivacaine (*n* = 7) or saline (*n* = 6) treatment prior to spinal nerve ligation. A sham (*n* = 3) group was also tested. Asterisks denote significant main effect of treatment detected by two-way ANOVA and Tukey’s posthoc analysis

Thermal hyperalgesia involves distinct pain processing that may be differentiated from mechanical allodynia [29, 30]. A subset of rats that were tested for mechanical allodynia were also tested for changes in thermal sensitivity before and following bupivacaine or saline application prior to SNL or sham treatment. There was no significant main effect of treatment [F(2,12) = 2.78; *p* = 0.10] or time post-injection [F(4,48) = 0.37; *p* = 0.83] on thermal hyperalgesia following SNL. In rats that received a 30-min perineural application of saline, there was no significant change in thermal thresholds in the ipsilateral hindpaw at any time points tested compared to sham controls (Fig. 3a). In rats that received a preemptive 30-min perineural application of bupivacaine, there was no significant change from basal thermal sensitivity in the ipsilateral paw (Fig. 3a). Furthermore, there was no significant main effect of treatment [F(2,11) = 0.97; *p* = 0.41] or time post-injection from baseline [F(4,44) = 0.92; *p* = 0.46] in the contralateral hindpaw (Fig. 3b).

**Fig. 4:**
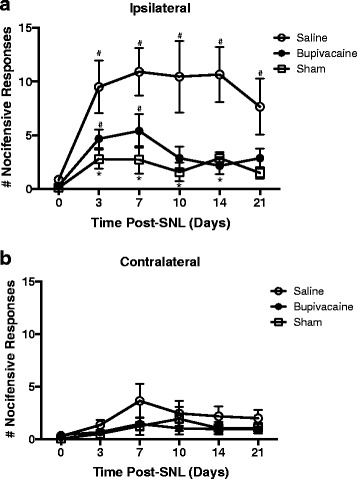
Preemptive bupivacaine attenuates acetone-induced cold allodynia. The number of shakes and/or licking episodes of the hindpaw following acetate application is reported as a measure of nocifensive behavior to a cold stimulus. Acetone applied to the ipsilateral (**a**) and contralateral (**b**) hindpaw. Responses were recorded prior to and 3, 7, 10, 14 and 21 days following bupivacaine (*n* = 8) or saline (*n* = 8) treatment prior to spinal nerve ligation. A sham (*n* = 5) group was also tested. Asterisks denote significant main effect of treatment detected by two-way ANOVA and Tukey’s posthoc analysis. * denotes significance from saline treated at *p* < 0.05; # denotes significance from baseline at *p* < 0.05

A separate group of rats was used to next examine the effect of preemptive bupivacaine on the incidence of neuropathic cold allodynia. Changes in cold sensitivity were detected as nocifensive behaviors following acetone application to the hindpaw. There was a significant main effect of treatment [F(2,18) = 6.39; *p* = 0.008] and time post-injection from baseline [F(5,90) = 8.05; *p* < 0.0001] on the degree of SNL-evoked cold allodynia. In rats that received a 30-min perineural application of saline at the L5 spinal nerve prior to nerve ligature, the ipsilateral hindpaw displayed significant cold allodynia within 3 days following spinal nerve ligation and lasting through 21 days compared to sham controls (Fig. 4a). In rats that received a preemptive 30-min perineural application of bupivacaine, cold allodynia was significantly attenuated at 10 and 14 days following SNL as compared to saline treated controls (Fig. 4a). Cold allodynia in rats treated with bupivacaine was not significantly different from sham controls at all time points tested (*p* > 0.05). Furthermore, there was a significant interaction of treatment over time [F(10,90) = 1.94; *p* = 0.05]. Cold allodynia was significantly greater than baseline measures on days 3 and 7 following SNL (*p* < 0.05), however the 10, 14 and 21 day time points were not significantly different from baseline (*p* > 0.05). There was a significant main effect of time post-injection [F(5,90) = 4.16; *p* = 0.002], but not treatment [F(2,18) = 1.20; *p* = 0.32] of cold allodynia in the contralateral hindpaw (Fig. 4b). However, there was no significant interaction of treatment over time in the contralateral paw [F(10,90) = 0.75; *p* = 0.68].

## Discussion

Pain associated with neuropathies is often difficult to manage effectively as the mechanisms involved in the initiation and maintenance of neuropathic pain remain unclear. Nerve damage evokes electrical hyperexcitability and abnormal nerve pulse generation that develops in primary sensory neurons with damaged afferents [31] and progresses to changes in spinal and supraspinal pain processing [8]. The consequence of these anatomical alterations could include the development of chronic pain conditions affecting both injured and uninjured areas through the process of central sensitization [32, 33]. The Neuropathic Pain Special Interest Group of the International Association for the Study of Pain released a consensus report recommending ion channel blockers as a first-line treatment for neuropathic pain [34].

Clinical studies have reported that local anesthetic ion channel blockers, such as lidocaine, may reduce neuropathic pain [35, 36]. Preemptive lidocaine has been reported to attenuate allodynia in a rat model of chronic constriction injury (CCI) [14] and partial transection neuropathy (PTN) [13], but not partial constriction neuropathy (PCN) [13]. However lidocaine is suboptimal due to short-acting duration of effect and possible systemic toxicity in large doses. This is problematic as nerve injury evokes a significant increase in spontaneous discharge in rat afferents within 24 h and is reduced within 5 days [37], however neuropathic pain behaviors often persist for weeks [38]. Furthermore, the effects of preemptive local anesthetic remain unclear in preclinical research due to the variability in findings between animal models of neuropathy.

In the present study, mechanical and cold allodynia developed within 3 days following ligation of L5 using the SNL model. Here we report that a single preemptive perineural application of bupivacaine initially did not block the onset of allodynia, but did attenuate mechanical and cold allodynia as early as 7–10 days following nerve injury. Mechanical allodynia was not significantly different from uninjured sham controls as early as 7 days following nerve injury, while cold allodynia did not differ from sham controls at all time points observed. Indeed, sham and bupivacaine treated rats were indistinguishable by 2 weeks post-SNL. In contrast, Abdi et al. [15] reported that local bupivacaine treatment was only effective at 24 h post-injury, but not at later time points up to 7 days. This discrepancy may be due to differences in severity of ligation (ligation of both L5 and L6 vs only L5) and/or timing of bupivacaine treatment (10 vs 30 min). Furthermore, the present study did not analyze neuropathic pain behaviors 24 h following injury. This is a limitation to the current study as it is possible that a transient effect could have occurred prior to 3 days post-injury. While no overt differences between the two variants of the SNL model have been reported, it is more probable that the duration of treatment accounts for this discrepancy and remains to be addressed in further studies. Regardless, our findings mirror findings that have been reported in clinical studies. In patients undergoing third molar extractions, intraoral preemptive bupivacaine prior to surgery did not have a significant effect on pain ratings at 24 h post-surgery, but bupivacaine treated patients did report lower pain intensities at 48 h post-surgery [20]. Similarly, our data showed that preemptive local bupivacaine did not block the initial development of pain behaviors, but did attenuate persistent allodynia.

Importantly, our data indicate that preemptive local bupivacaine significantly shortened recovery time from SNL-induced mechanical and cold allodynia, indicating that bupivacaine may block the occurrence of some persistent neuropathic pain conditions. These data are also in agreement with a previous study reporting efficacy of preemptive bupivacaine in a different nerve injury model, the rat spared nerve injury (SNI) model [39]. Together, these studies provide preclinical evidence in two distinct neuropathic pain models that preemptive perineural bupivacaine improves neuropathic pain outcomes. Thus preemptive bupivacaine appears to be effective in some neuropathic pain models and not others in reducing allodynia, mirroring opposing findings in clinical studies. Further studies to determine whether preemptive bupivacaine is effective in animal models of chemotherapy-induced neuropathic pain (CINP) or post-surgical pain, and as compared to other local anesthetics, are warranted.

Bupivacaine could be acting via (1) reducing ectopic firing activity in primary afferent fibers, (2) reducing central sensitization, and/or (3) altering descending facilitation, which is known to regulate the maintenance of pain rather than its onset [8, 12]. Research indicates discrete processes are involved in the *onset* versus *maintenance* of neuropathic pain. Burgess et al. [25] reported that the initiation of neuropathic pain (days 0–3) is independent of, but maintenance (days 6–12) is modulated by, supraspinal influences. In support, ligation of serotonergic descending pathways in the SNL model does not affect the onset of mechanical hypersensitivity, but does attenuate mechanical hypersensitivity one week later (see review [8]). Thus one hypothesis is that preemptive bupivacaine is acting in a manner that is preventing supraspinal facilitation of neuropathic pain. This hypothesis is supported by the differential effects of preemptive bupivacaine on the onset versus maintenance of allodynia in the present study. Further studies examining relative alterations in ion channel expression and electrophysiological or neurographical recordings are warranted to provide insight into what anatomical level(s) is being altered at 3 versus 7 days following preemptive bupivacaine and nerve injury.

Xie et al. [12] reported that nerve blockade immediately following injury and lasting 3–5 days significantly attenuated allodynia in the chronic constrictive injury (CCI) and spared nerve injury (SNI) rat models of neuropathic pain. In support, intercostal bupivacaine administered prior to rat thoracotomy prevents the development of pain behaviors in a greater number of animals than post-surgical administration or when given systemically before surgery [40]. This has also been observed clinically in patients undergoing third molar surgery [41] and knee ligament reconstruction [42]. One clinical trial was able to compare preincisional to postincisional treatment with mepivacaine and reported a significant reduction in postoperative pain at 6 months [43]. However studies have also reported no differences in pain outcomes following pre- versus postoperative treatment [44].

In the present study, SNL did not induce significant thermal hyperalgesia when comparing SNL and sham groups while mechanical and cold sensitivity were robust, in agreement with some studies [45–47] and discordant with others [12, 22, 25]. The reasons for this difference are not discernable from the present study, but are likely due to differences in weight-bearing of the injured paw [45], test-dependent magnitude of thermal sensitivity [29, 30, 48], and/or potential differences in temporal presentation of thermal hyperalgesia or *hypo*algesia [45, 49]. Despite these differences, the presence of significant mechanical and cold allodynia provides a clinically relevant neuropathic pain indicator in the present study.

There are a variety of therapeutics targeting different pain mechanisms that are used in clinical settings, however there is no one treatment that has proven effective for neuropathic symptoms in all patients. Anticonvulsants such as gabapentin are also recommended [34], presumably due to their ability to reduce calcium channel conductance. Gabapentin has been reported to be effective for pain associated with various peripheral neuropathies in clinical trials [50, 51], but requires several weeks of treatment before an attenuating effect is observed. In preclinical studies, continuous infusion of gabapentin for 7 days following injury was effective in attenuating mechanical allodynia in rats [52]. Furthermore, perineural pregabalin treatment produced superior analgesia compared to systemic administration [53].

Other ion channel blockers may also be effective for the preemptive treatment of neuropathic pain. Blockade of early spontaneous afferent activity with the sodium channel blocker tetrodotoxin (TTX) following SNL also suppresses neuropathic pain [12]. The blockade of signaling by TTX was accompanied by reduced activation of satellite glial cells in the dorsal root ganglia (DRG), which could explain its analgesic effect as activated glial cells play a central role in neuropathic pain [54, 55]. Ketamine exerts at least part of its analgesic effects through antagonism of the N-Methyl-D-aspartate (NMDA) receptor. As both a glutamate receptor and a calcium ion channel, ketamine’s actions on NMDA receptors may be an effective preemptive analgesic for neuropathic pain [56, 57]. Indeed, preemptive intrathecal ketamine attentuates allodynia for 2 weeks following nerve injury [58].

## Conclusions

The postoperative pain that results from surgical nerve damage has a profound impact on recovery and quality of life and is a major clinical problem [8]. Our data provide preclinical evidence supporting the use of local preemptive bupivacaine to attenuate the persistent occurrence of allodynia following surgical manipulations that are known to cause nerve damage, such as invasive surgery, nerve dissection and amputations in the military Service Member and civilian populations. The use of preemptive local bupivacaine in patients undergoing surgery, for example through ultrasound-guided paravertebral injection [59], may shorten the recovery from nerve injury-evoked mechanical and cold allodynia.
